# The evolution of IBD perceived engagement and care needs across the life-cycle: a scoping review

**DOI:** 10.1186/s12876-021-01850-1

**Published:** 2021-07-14

**Authors:** E. Volpato, C. Bosio, E. Previtali, S. Leone, A. Armuzzi, F. Pagnini, G. Graffigna

**Affiliations:** 1 Department of Psychology, Università Cattolica del Sacro Cuore, Largo A. Gemelli, 1, 20123 Milan, Italy; 2 IRCCS Fondazione Don Carlo Gnocchi, Milan, Italy; 3 EngageMinds Hub Consumer, Food and Health Research Center, Università Cattolica del Sacro Cuore, Milan, Cremona, Italy; 4 Department of Psychology, Harvard University, 33 Kirkland Street, Cambridge, MA 02138 USA; 5 AMICI Onlus, Associazione nazionale per le Malattie Infiammatorie Croniche dell’Intestino, Milan, Italy; 6 Università Cattolica del Sacro Cuore di Roma, Rome, Italy; 7 IRCCS Policlinico Gemelli, Rome, Italy

**Keywords:** Inflammatory Bowel Disease (IBD), Patient engagement, Medical needs, Psychosocial needs

## Abstract

**Background:**

The chronic and progressive evolution of Inflammatory Bowel Diseases (IBD), with its prototypical fluctuating trend, creates a condition of psycho-social discomfort, impacting the quality of life in terms of personal, working, and interpersonal.

**Aims:**

In this article, we want to identify the nature and extent of the research evidence on the life experiences, the perceived engagement, the psychological, social care and welfare needs of people affected by IBD across the lifecycle.

**Methods:**

Following the approach set out by Arksey and O’Malley and the PRISMA extension for scoping reviews, we conducted a scoping review in March 2019 and closed the review with an update in October 2019. It was performed using electronic databases covering Health and Life Sciences, Social Sciences and Medical Sciences, such as PubMed, Medline, Embase, Scopus, Cochrane, Web of Science, PsycInfo.

**Results:**

We identified 95 peer-reviewed articles published from 2009 to 2019, that allowed to detection the main needs in children (psychological, need to be accepted, physical activity, feeding, parent style, support, social needs), adolescents (to understand, physical and psychological needs, protection, relational, gratitude, respect, and engagement) and adults (information, medical, psychological, social, work-related, practical, future-related, engagement). Although the literature confirms that the majority of the IBD units have planned provision for the different types of transitions, the quality and appropriateness of these services have not been assessed or audited for all the kinds of challenges across the life cycle.

**Conclusions:**

The literature shows the relevance of organizing a flexible, personalized health care process across all the critical phases of the life cycle, providing adequate benchmarks for comparison in a multidisciplinary perspective and ensuring continuity between hospital and territory.

**Supplementary Information:**

The online version contains supplementary material available at 10.1186/s12876-021-01850-1.

## Background

Inflammatory Bowel Disease (IBD) is a group of idiopathic, chronic, and relapsing clinical conditions that primarily affect the gastrointestinal tract. IBD includes two different chronic diseases, Ulcerative Colitis (UC) and Crohn’s Disease (CD), closely related but with heterogeneous disease processes. Worldwide, the prevalence of IBD is estimated to reach up to 396 people per 100,000 with the highest incidence reported in North America, Northern and Western Europe [1]. It is currently estimated that more than 3 millions people in Europe and more than 5 million worldwide suffer from IBD [2]. There is a rising trend, attested that 75% of CD studies and 60% of UC studies stress a rapidly increasing incidence. Considering UC, prevalence rates of up to 505 and 249 people per 100,000 individuals are recorded, respectively in Europe and North America [3]. In general, the peak age of onset for CD is between 20 and 30 years of age, whether for UC is between 30 and years old [4, 5]. Urbanity and rapid industrialization in the Eastern World seem to be related to this trend [3]. In particular, among the causes of this build-up, were also listed milk formula feeding, use of appendectomy, lifestyle, and changing diets [6].

In the specific case of UC, the episodes of remission and relapse are unpredictable and highly variable from one patient to another: some patients experience prolonged asymptomatic periods and those who continually face the symptoms of UC [7, 8]. UC often occurs in adolescence and young adulthood and is characterized by the presence of diarrhea, flatulence, fecal urgency, abdominal pain and cramps, rectal bleeding and fatigue, symptoms that negatively impact on Quality of Life (Quality of Life, QoL) and the well-being of the person [9]. CD, on the other hand, in addition to causing abdominal pain, distension, swelling, and vomiting, if restrictive it may precede the development of an internal disease with the formation of fistulas [10], significantly impacting the QoL. Previous studies highlight that also psychological issues may play a negative role in disease activity and QoL [11, 12]. Nevertheless, it is still unclear if and, eventually, how psychological factors can also contribute to the development of people with IBD [13].

Growing attention in the last decade has been paid to the role of IBD patients in their care management [14, 15]. To enhance IBD patients’ participation along their patient journey it is important to understand their care needs and expectation. Improving psychological counseling, social welfare and care and the overall quality of the medical and pragmatic assistance provided to IBD patients is an urgent goal for bettering patients’ wellness and quality of life [16].

The debate about IBD patients care needs and priority is growing although fairly fragmented. Searle and Bennet [17] reviewed a decade of psychological literature debate to synthesize the relationship between psychological stress and IBD symptomatology as well as to critically investigate the impact of psychological intervention on IBD symptoms management. Graff et al. [18] have systematized the evidences of psychological needs in IBD care by discussing the importance of enhancing psychological counselling to sustain the effectiveness of clinical interventions. Kemp et al. [19] conducted a meta-synthesis of qualitative research evidence about IBD patients’ care needs by systematizing the psychological tensions and conflicts experienced during daily life between the management of the disease symptoms and the attempt to keep some form of life normality. McCombie et al. [20] reviewed copying strategies of IBD patients and underlined priorities for psycho-therapy and psychological intervention in synergy with medical care. Psychological needs, social care needs, assisting with activities of daily living, maintaining independence, social interaction, enabling the individual to play a fuller role in society, protecting them in vulnerable situations, helping them manage complex relationships, and (in some circumstances) accessing a nursing home or other supported housing, as well as social welfare needs, that can be defined as the set of assistance programs designed to ensure the well-being of a nation's citizens, turn out to be very relevant in the management of IBD. However, recent reviews or classifications of the literature about IBD psychological and social care and welfare needs are lacking. Furthermore, the available reviews are mainly focused on the psychological needs of IBD patients and do not considers their pragmatic and social care and welfare needs, which are important in the goal of increasing patients’ engagement in self-management. Finally, the reviews available in the literature mainly focus on adult patients, whereas less attention has been paid to pediatric patients and in particular to their transition to adulthood.

In order to fill these gaps and improving social welfare care and patient engagement initiatives dedicated to IBD patients along their life course, we conducted this scoping review. More into details, this scoping review aimed to identify and describe the evidence on life experiences, psychosocial and social needs in people with IBD. Psychological needs are essential to mental health, and they can be generated either internally or by interactions between the individual and the environment. The fluctuating nature of people with IBD has been described to damage employment, social and leisure activities, relationships, and psychological well-being arising a different kind of needs. This review provides the basis for developing broader research on the relatively underexplored topics and consequently improves specific interventions that could address patients’ needs.

### Objectives

Based on the premise, this scoping review was conducted with the main objective of identifying the key concepts that characterize the psychological, social care, and welfare needs of people affected by IBD across the lifecycle by highlighting the main types of published evidence and sources available. In this case, the scoping review was carried out to explore previous research activity, disseminate findings, and identify gaps in the comprehension of patient’s needs. Moreover, a secondary objective was to explore factors that increase patient engagement.

## Methods

This scoping review was set out thanks to the approach based on the five-stage framework created by Arksey and O’Malley and the PRISMA extension for scoping reviews [21]. The five stages are as follows: identifying the initial research questions; identifying relevant studies, study selecting; charting the data; collating, summarizing and reporting the results. This approach guarantees the implementation of a rigorous and transparent process, which increases the reliability and replicability of the study findings [22].

### Identifying the initial research question

According to the objectives (cfr. par. 1.1), this review was guided by the following research questions:

- What are the overriding psychological and social care and welfare needs of people with IBD in the patient journey?
  - What are the overriding psychological and social care and welfare needs in children with IBD?
  - What are the overriding psychological and social care and welfare needs in adolescents with IBD?
  - What are the overriding psychological and social care and welfare needs in adults with IBD?
- How do the psychological and social care and welfare needs evolve along the IBD patient life cycle?
  - What are the needs that emerge during childhood transitions?
  - What needs emerge during adolescent-age transitions?
  - What are the needs that emerge during adulthood transitions?
- What are the factors which may increase IBD patient engagement in their patient journey?
  - What are the factors which may increase IBD patient engagement in children?
  - What are the factors which may increase IBD patient engagement in adolescents?
  - What are the factors which may increase IBD patient engagement in adults?

### Identifying relevant studies: the literature search

The literature search was conducted from March to October 2019, using electronic databases covering Health and Life Sciences, Social Sciences, and Medical Sciences, such as PubMed, Medline, Embase, Scopus, Cochrane, Web of Science, PsycInfo. In addition, a hand search of the reference list of identified articles and reviews were undertaken as well as the “cited articles” search tool was used to identify any other primary sources within grey literature.

According to Arksey and O’Malley [22], key concepts and search terms were developed to catch available literature related to psychological, medical, and social care and welfare needs in people with IBD. The use of search tools such as educational subject headings and Boolean operators to narrow, widen and combine literature searches was used (Additional file 1: Appendix S1). Based on a preliminary review of the literature and our clinical and research experience, we formulated queries on three items: -the kind of IBD (keywords like ("IBD*" OR "inflammatory bowel disease*" OR "Crohn*" OR "ulcerative colitis"); -the kind of need (keywords like ("Psych*" OR "social*" OR "famil*" OR "engagement" OR "empowerment"); -the kind of possible strategy to cope with the need (keywords like "intervention" OR "trial" OR "program" OR "strateg*" OR "counsel*" OR "treatment" OR "support”). The keyword used developed to guide the search are outlined in Table 1.

**Table 1:** Key-words adopted for the search strategy

Key-words	
("IBD*" OR "inflammatory bowel disease*" OR "Crohn*" OR "ulcerative colitis") AND ("Psych*" OR "social*" OR "famil*" OR "engagement" OR "empowerment") AND ( "intervention" OR "trial" OR "program" OR "strateg*" OR "counsel*" OR "treatment" OR "support”)	

#### Inclusion and exclusion criteria

Inclusion and exclusion criteria were developed to be as comprehensive as possible in the identification of the evidence (Table 2).

**Table 2:** Inclusion and exclusion criteria

	Inclusion criteria	Exclusion criteria
Time period	2009–2019	Studies outside these dates
Language	English (recognized language of international scientific debate)	Non-English
Type of article	Original research published in a peer review journal. Qualitative or quantitative studies; commentaries, letter, editorial	Articles that were not peer reviewed, only abstract available
Ethics clearance	Studies with approved ethics notification	Studies without approved ethics notification
Study focus	Transitions during the life cycle and therefore in the passages between childhood and adolescence, adolescence and young adulthood, young adulthood and adulthood, adulthood and mature age, mature age and old age	Studies that don't consider transitions during the life cycle
Literature focus	Studies that explicitly discuss socio-assistance and psychological needs, good practices for social assistance, the concept of Patient Engagement, studies on social and/or psychological strategies that enhance the patient's point of view and his experience, studies that enhance the patient's point of view and experience, studies that present a clear theoretical framework on the patients' experience, studies that focus on the experience of using the treatment, quality of life indicators and monitoring, in the context of chronic pathologies considered	Articles that didn't make a passing or token reference to engagement and psychological and/or social welfare needs. Articles that were discussion or personal opinion pieces
Population and sample	Inflammatory Bowel Disease, IBD; Ulcerative Colitis, UC; Crohn’s Disease, CD	All the other chronic diseases

### Study selection

**Fig. 1:**
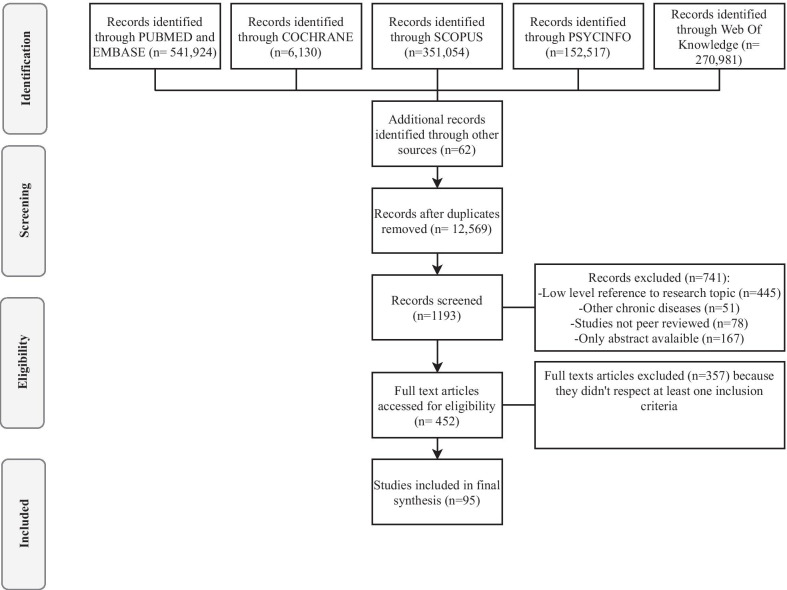
PRISMA flow diagram for article selection

Using the key search descriptors, in the different electronic databases, articles were identified, and duplicates were excluded (n = 12,569). After a first screening based on title and abstract, the records examined were 1193. An accurate review of both titles and articles allowed to reveal a large number of papers that showed low-level reference to research questions (n = 445) or studies related to other chronic diseases (n = 51). Moreover, other articles were excluded because they were not peer-reviewed (n = 78) or only the abstract was available (n = 167). Guided by the inclusion and exclusion criteria, 95 research articles were identified as being relevant to the research topic. The full version of the texts was considered, and each paper was reviewed and confirmed to be or not appropriate by the authors (EV; CB). Moreover, this process allowed us to consider the reference lists of each article and identify any further additional relevant literature. The process of article selection followed the PRISMA extension for scoping reviews, as mentioned above [21]. Figure 1 illustrates the selection process.

During the process of selection, several studies were excluded because they didn’t reflect the eligibility criteria.

### Data charting and collation

Arksey and O'Malley's delineated a fourth passage [22] that aims to chart the selected articles. Data were extracted using standardized forms and entered Microsoft Excel (Microsoft, Redmond, Washington, USA) by one reviewer (EV) and a 10% sample was checked for accuracy and completeness by another (FP). Reviewers resolved discrepancies through consensus. Given the qualitative and quantitative nature of the included studies, a structured analysis grid according to mixed methods approach was followed: (1) methodological characteristics of the study (e.g. year of publication, country of the first author, study design, number of participants); (2) characteristics of the participants (e.g. diagnosis, age group, presence of caregivers); (3) characteristics of socio-welfare and psychological needs (e.g. for which transition phase, type of need expressed, theoretical framework considered) 4) characteristics of the social and/or psychological strategy (if present: type of strategy, tools used, group or individual, theoretical foundations of the same); 5) results obtained (e.g. measured outcomes, method of evaluating results, overall results achieved). Details of the included studies are shown in Additional file 2: Table S2.

## Findings

This review yielded 95 articles from 22 countries. Most of them were conducted in the USA (n = 24, 25.7%) or in the UK (n = 15, 15.8%), followed by Canada (n = 8, 8.4%). Twenty-three articles (24.21%) were published in 2018, followed by 18 (18.9%) published in 2017, 14 (14.73%) in 2016, and 11 (11.6%) in 2015, indicating an increasing trend. A relevant part of the included studies, twenty-four (25.7%) were surveys and cross-sectional surveys. The most part were quantitative studies (n = 65, 68.4%) 8 (8.4%) studies were qualitative, 6 (6.3%) were mixed-method studies and 19 (29.2%) were reviews or guidelines.

Sixty articles (63.15%) are related to needs in adults, 56 (58.9%) are inherent to transitional needs, 68 (71.5%) regard needs in adolescents, and 20 (21.05%) in children. A total of 23,776 participants with an age range of 31.55 were included. It is relevant to note that most articles cover more than one area of need (Table 3). Fifty studies (52.6%) focus on IBDs in general without any sort of specification, 4 (4.2%) are related to UC, and 7 (7.3%) regard CD. The others remaining specified clearly how many patients had what kind of diagnosis.

**Table 3:** Articles included in the review and divided considering the main theme and phase of the life cycle

	Number of records	References
*Children*		
Psychological needs and need to be accepted	3	Zmeskalova et al., 2016; Bokemeyer et al., 2013; Dignass et al., 2015
Needs related to physical needs and physical activity	2	Mählmann et al., 2017; Vedrana, Bramhagen, Idvall, and Wennick, 2017
Needs related to feeding	10	Pituch-Zdanowska et al., 2019; Miele et al., 2018; Rocha, Sousa, Reis, and Santana, 2019; Tomar, Sanjeevani K; Kedia, Saurabh; Upadhyay, Ashish D, Bopanna, Sawan et al., 2017; Cohen, Aaron B; Lee, Dale; Long, Millie D; Kappelman, Michael D., et al., 2014; Hou, Jason K; Lee, Dale; Lewis, James, 2014; De Vries, Jeanne H.M.; Dijkhuizen, Milou; Tap, Petra Witteman, Ben J.M., 2019; Kakodkar, Samir, Mutlu, Ece A, 2017
Parenting style and support	3	Kluthe et al., 2018; Lindström, Åman, Anderzén-Carlsson, and Lindahl Norberg, 2016; Zmeskalova et al., 2016
Social needs	2	Vedrana et al., 2017; Vejzovic, Bramhagen, Idvall, and Wennick, 2018
*Adolescents*		
Need to understand	9	Olsen, Jensen, Larsen and Sorensen, 2016; Gumidyala et al., 2017; Cho et al., 2018; Cho et al., 2018; Fu et al., 2017; McCartney, 2011; Gumidyala et al., 2018; Gray et al., 2015; Klostermann, McAlpine, Wine,Goodman and Kroeker, 2018
Physical needs	3	Olsen, Jensen, Larsen and Sorensen, 2016; Zmeskalova et al., 2016; Smith and Gettings, 2016
Psychological needs	8	Gamwell et al., 2018; Wu and Zhong, 2018; Zmeskalova et al., 2016; Smith and Gettings, 2016; Cho et al., 2018; Cho et al., 2018; Hummel et al., 2013; Lugasi, Achille and Stevenson, 2011
Need of protection, safeness	3	Gray et al., 2018, 2015; Reiss, Gibson, and Walker, 2017; Smith and Gettings, 2016
Relational and social needs	13	Hummel et al., 2013; Vedrana et al., 2017; Adler and Sc, 2016; Olsen, Jensen, Larsen and Sorensen, 2016; Gamwell et al., 2018; Wu and Zhong, 2018; Zmeskalova et al., 2016; Cho et al., 2018; Cho et al., 2018; De Silva and Fishman, 2014; Rohatinsky, Risling, Kumaran, Hellsten and Thorp-Froslie, 2018; McCartney, 2011; Gumidyala et al., 2018; Gray et al., 2015
Need for gratitude, respect and respect for your needs	1	Olsen, Jensen, Larsen and Sorensen, 2016
Engagement needs	31	Stollon et al., 2017; Zeisler and Hyams, 2014; Rosen, Annunziato, Colombel and Benkov, 2015; Cho et al., 2018; Plevinsky, Gumidyala and Fishman, 2015; Gray et al., 2018; Yerushalmy-Feler et al., 2017; Escher, 2009; van Groningen, Ziniel, Arnold and Fishman, 2012; Karim, Porter, McCombie, Gearry and Day, 2019; Timmer et al., 2017; Adler, 2016; Szeto et al., 2018; Fishman, Mitchell, Lakin, Masciarelli and Flier, 2016; Fu et al., 2017; Fishman, Barendse, Hait, Burdick and Arnold, 2015; Jeganathan et al., 2017; De Silva and Fishman, 2014; Rohatinsky, Risling, Kumaran, Hellsten and Thorp-Froslie, 2018; McCartney, 2011; Bennett, Moore, Bampton, Bryant and Andrews, 2016; Otto et al., 2019; Cole et al., 2015; Gumidyala et al., 2018; Maddux, Ricks and Bass, 2017; Schmidt, Herrmann-Garitz, Bomba and Thyen, 2016; Gray et al., 2015; Gray et al., 2015; Van den Brink et al., 2019; Klostermann, McAlpine, Wine,Goodman and Kroeker, 2018; Lugasi, Achille and Stevenson, 2011
*Adults*		
Needs of information	11	Wu and Zhong, 2018; Butcher, Law, Prudham and Limdi, 2011; Bernstein, Promislow, Rawsthorne, Walker and Bernstein, 2011; Catalan-Serra et al., 2015; Gordon, Mcewan, Maguire, Sugrue and Puelles, 2015; McDermott et al., 2018; van Deen et al., 2017; Wardle and Mayberry, 2014; Baker, Lee, Jones, Brown and Lobo, 2018; Kamp and Brittain, 2018; Mikocka-Walus, Power, Rook and Robins, 2015
Medical needs	1	Baker DM et al., 2018
Psychological needs	13	Jordan, Ohlsen, Hayee and Chalder, 2018; Knowles, Andrews and Porter, 2018; Klag et al., 2017; Goodhand, Wahed and Rampton, 2009; Chiarini et al., 2017; van Erp et al., 2017; Mcdermott et al., 2015; Tribbick et al., 2017; Vegni et al., 2018; Muller, Prosser, Bampton, Mountifield and Andrews, 2010; Wang et al., 2016; Trindade, Ferreira and Pinto-Gouveia, 2017; Gurková and Soósová, 2018
Social and work related needs	8	Calvet et al., 2018; Leso, Ricciardi and Iavicoli, 2015; Restall et al., 2016; Schreiner et al., 2017; van der Have et al., 2015; Haapamäki, Turunen, Roine, Färkkilä and Arkkila, 2009; Froes et al., 2018; Muller, Prosser, Bampton, Mountifield and Andrews, 2010
Support needs	10	Jordan, Ohlsen, Hayee and Chalder, 2018; Garcia-Sanjuan, Lillo-Crespo, Sanjuan-Quiles, Gil-Gonzalez and Richart-Martinez, 2016; Knowles, Andrews and Porter, 2018; Lahat, Neuman, Eliakim and Ben-Horin, 2014; Chiapponi, Witt, Dlugosch, Gulberg and Siebeck, 2016; Klag et al., 2017; Goodhand, Wahed and Rampton, 2009; Iglesias et al., 2009; Dibley et al., 2018; Kamp and Brittain, 2018
Practical needs	11	Limdi, Aggarwal and McLaughlin, 2016; Limdi, 2018; Kakodkar and Mutlu, 2017; De Vries, Dijkhuizen, Tap and Witteman, 2019; Larussa et al., 2019; Hou, Lee and Lewis, 2014; Cohen et al., 2014; Rocha, Uli, Reis and Santana, 2019; Tomar et al., 2017; Albenberg et al., 2019; Schreiner et al., 2019
Needs related to the future	2	Nutting and Grafsky, 2018; Ghorayeb, Branney, Selinger and Madill, 2018
Engagement needs	5	Dibley et al., 2018; Kamp and Brittain, 2018; Siegel et al., 2016; Mikocka-Walus, Power, Rook and Robins, 2015; Carpio et al., 2016
*Transitions*		
Transition to school	0	
Transition to adolescence	2	Cullen and Beattle, 2009; Fu et al., 2017
Transition to adulthood	46	van Groningen, Ziniel, Arnold and Fishman, 2012; Cullen and Beattle, 2009; Van den Brink et al., 2019; Stollon et al., 2017; Yerushalmy-Feler et al., 2017; Timmer et al., 2017; Gray et al., 2018; Plevinsky, Gumidyala and Fishman, 2015; Cho et al., 2018; Zeisler and Hyams, 2014; Rosen, Annunziato, Colombel and Benkov, 2015; Karim, Porter, McCombie, Gearry and Day, 2019; Adler, 2016; Szeto et al., 2018; Fishman, Mitchell, Lakin, Masciarelli and Flier, 2016; Fu et al., 2017; Fishman, Barendse, Hait, Burdick and Arnold, 2015; Jeganathan et al., 2017; De Silva and Fishman, 2014; Rohatinsky, Risling, Kumaran, Hellsten and Thorp-Froslie, 2018; McCartney, 2011; Bennett, Moore, Bampton, Bryant and Andrews, 2016; Otto et al., 2019; El-Matary, 2009; Elli et al., 2019; Clarke, Tilean, Lusher and Joanne, 2016; Afzali and Wahbeh, 2017; Bincy and Stacy, 2014; van Rheenen et al., 2017; Brooks et al., 2017; Brooks, Smith and Lindsay, 2018; Cole et al., 2015; Shapiro et al., 2019; Leung, Heyman and Mahadevan, 2011; Gumidyala et al., 2018; Hummel et al., 2013; Maddux, Ricks and Bass, 2017; Bollegala and Nguyen, 2015; Schmidt, Herrmann-Garitz, Bomba and Thyen, 2016; Gray et al., 2015; Gray et al., 2015; Van den Brink et al., 2019; Trivedi, Holl, Hanauer and Keefer, 2016; Klostermann, McAlpine, Wine,Goodman and Kroeker, 2018; Lugasi, Achille and Stevenson, 2011; Goodhand, Hedin, Croft and Lindsay, 2011;
Transition during the adulthood	3	Gordon, Mcewan, Maguire, Sugrue and Puelles, 2015; Ghorayeb, Branney, Selinger and Madill, 2018; Nutting and Grafsky, 2018
Transition to remissions	1	Iglesias et al., 2009
Transition to/from surgery	4	Olsen, Jensen, Larsen and Sorensen, 2016; Kamp and Brittain, 2018; Dibley et al., 2018; Baker, Lee, Jones, Brown and Lobo, 2018
Transition to ageing	0	
No transition	53	Smith and Gettings, 2016; Gumidyala et al., 2017; Zmeskalova et al., 2016; Gamwell et al., 2018; Wu and Zhong, 2018; Butcher, Law, Prudham and Limdi, 2011; Carpio et al., 2016; Bernstein, Promislow, Rawsthorne, Walker and Bernstein, 2011; Catalan-Serra et al., 2015; McDermott et al., 2018; van Deen et al., 2017; Wardle and Mayberry, 2014; Jordan, Ohlsen, Hayee and Chalder, 2018; Garcia-Sanjuan, Lillo-Crespo, Sanjuan-Quiles, Gil-Gonzalez and Richart-Martinez, 2016; Knowles, Andrews and Porter, 2018; Lahat, Neuman, Eliakim and Ben-Horin, 2014; Chiapponi, Witt, Dlugosch, Gulberg and Siebeck, 2016; Klag et al., 2017; Calvet et al., 2018; Froes et al., 2018; Leso, Ricciardi and Iavicoli, 2015; Restall et al., 2016; Schreiner et al., 2017; van der Have et al., 2015; Siegel et al., 2016; Limdi, Aggarwal and McLaughlin, 2016; Limdi, 2018; Kakodkar and Mutlu, 2017; De Vries, Dijkhuizen, Tap and Witteman, 2019; Larussa et al., 2019; Schreiner et al., 2019; Hou, Lee and Lewis, 2014; Cohen et al., 2014; Rocha, Uli, Reis and Santana, 2019; Tomar et al., 2017; Chiarini et al., 2017; van Erp et al., 2017; Gurková and Soósová, 2018; Mcdermott et al., 2015; Tribbick et al., 2017; Vegni et al., 2018; Muller, Prosser, Bampton, Mountifield and Andrews, 2010; Wang et al., 2016; Trindade, Ferreira and Pinto-Gouveia, 2017; Mikocka-Walus, Power, Rook and Robins, 2015; Albenberg et al., 2019; Pituch-Zdanowska et al., 2019; Miele et al., 2018; Haapamäki, Turunen, Roine, Färkkilä and Arkkila, 2009; Goodhand, Wahed and Rampton, 2009
No needs	13	El-Matary, 2009; Elli et al., 2019; Clarke, Tilean, Lusher and Joanne, 2016; Afzali and Wahbeh, 2017; Bincy and Stacy, 2014; van Rheenen et al., 2017; Brooks et al., 2017; Brooks, Smith and Lindsay, 2018; Shapiro et al., 2019; Leung, Heyman and Mahadevan, 2011; Bollegala and Nguyen, 2015; Trivedi, Holl, Hanauer and Keefer, 2016; Goodhand, Hedin, Croft and Lindsay, 2011

Participants are recruited from IBD clinics, hospitals, outpatients’ clinics, national charities, community-based study, and media advertisements.

We resumed in the following sections the main findings related to our research questions (cfr. par. 2.1).

Additional file 3: Table S3 shows the differences in care and needs divided considering the main theme and phase of the life cycle.

### The needs in children with IBD

The main needs in children with IBD cover five areas.

#### Psychological needs and need to be accepted

Children may express anxiety and, sometimes, distress, because of the chronic symptoms or exacerbations experienced. In general, the needs of a child tend to be concerning those expressed by the parents and the pediatrician who takes care of them, since everyone has the unconscious desire to completely disappear the symptoms. In the specific case of children, tolerance, empathy, hope, safety, and control, need to be carefully managed also towards parents and pediatricians [23]. Psychological problems such as depression, helplessness, anxiety, family arguments, and school difficulties have not been recognized as associated with IBD [24]. Symptoms related to anxiety and depression tend to be lower in children than, for example, in adults with IBD. However, evidence shows that few studies to date have accurately explored these issues and have done so with different tools, which is why further investigation is needed. Similarly, non-specific symptoms such as, for example, fatigue, dizziness, headache, frequent infections, or shortness of breath, were not considered associated with IBD [25].

#### Needs related to physical needs and physical activity

Children report the desire to feel healthy and tend to be afraid to talk about their pathology. They say that they can only rarely share with their parents how they feel physically and their thoughts because they are afraid of raising further concerns. Furthermore, the changes in one's body that could occur if it were necessary to remove the intestine provoke fear. In particular, they are embarrassed if they have to confide in someone they need to often resort to the toilet, so they tend not to talk about it with anyone. For this reason, they often skip physical activities [26, 27].

#### Needs related to feeding

Studies stress how, due to their dietary beliefs, parents of children with IBD, commonly introduce dietary restrictions. Despite malnutrition and specific nutritional deficiencies are frequent among IBD patients, and the majority of them need nutritional treatment, there are no evidence for an answer to this need in children [28, 29]. Moreover, only a few studies have considered nutritional status as a risk factor associated with higher rates of hospitalization and the time passed at a clinic [30]. Miele and colleague tried to defined the steps in instituting dietary or nutritional management in light of the current evidence and to offer a useful and practical guide to physicians and dieticians involved in the care of pediatric IBD patients, paying peculiar attention to the alimentary options available (e.g. Exclusive Enteral Nutrition (EEN); Partial Enteral Nutrition (PEN); Specific carbohydrate diet (SCD); Lactose-free diet (LFD); Crohn’s disease exclusion diet (CDED)) [31].

#### Parenting style and support

The type of parenting style is important: indeed, scientific literature has shown that one of the parents, usually the mother, tends to be overprotective towards the children, facilitating the development of the “perpetually sick child”, which would significantly obstacle responsibility’s development. While both parents may show significant fear and/or apprehension about their child's health, usually the responsibility for most home and health care activities is placed on the mother. However, there are very few studies about the role of parents in both the infant and adolescent care processes [23, 32, 33]. In addition, children with IBD have an increased risk of behavioral and family dysfunction, as well as nonadherence to treatment regimens. However, there are still very few and heterogeneous data available, especially if considering transitions, to draw firm conclusions [34]. The reason for this scarcity of literature could be related to the fact that, with a child affected by a chronic disease, parents tend physiologically to be more apprehensive and have difficulty managing normal boundaries. Parents reported a change in how they responded to their children's health and physical ability after diagnosis. While some parents feel they don't have to insist as much as they did before diagnosis, others actively encourage their children to be as active as possible. Parents are also often concerned about how their children are doing compared to their friends, or a baseline considered “healthy” or “normal”. Furthermore, the majority of parents after the diagnosis are concerned with the preparation and consumption of food with greater attention than in the past. Whether it is supporting your children by moving the whole family on a special diet or by making an effort not to eat foods that your child cannot take. Discussion of parenting techniques in this context can be helpful [23, 32, 33].

#### Social needs

A large number of articles reported that children with IBD experience fear of being excluded or feelings of deep loneliness, sometimes preferring this latter, because they are afraid that others perceive them as “sick” or “different”. For this reason, while appreciating the help of teachers and nurses at school, they tend not to often ask for help, because they live it with embarrassment or humiliation [27]. The children indicate that they have some close friends, with whom they can share their thoughts and “talk about everything”. However, it seems that they are more likely to talk about their condition in general, avoiding referring to specific physical states attributable to the pathology. In addition, many of them report having more pleasure in sharing how they feel and their thoughts with those who live in the same condition because they are perceived as more able to understand their feelings and experiences [35]. Additional resources noted that the children feel themselves as healthy, but they are afraid of not being able to control the need for “triggers”, especially during school hours. The constant fear that something unpleasant may happen limits them in their daily life and induces fear of being excluded. In this situation, they are also concerned that they will no longer receive future invitations from the group of classmates due to frequent absences [27].

### The needs in adolescents with IBD

Researches highlight a significant misalignment in the estimation of health concerns between IBD adolescent patients and their pediatricians [36] (Additional file 2: Tables S2, Table 3).

#### Need to understand

Younger patients are often characterized by poor basic knowledge of their medical condition and possible treatments. Among the most relevant knowledge to be transmitted are the basic medical history and the nature of the clinical condition, its duration, the names and doses of drugs, allergies, the names of the medical team, and how to contact the team in case of need [37]. The reactions to the diagnosis are described by the adolescents as different according to the previous degree of knowledge of pathology itself: indeed, those who have previous knowledge due to family experiences or readings about the pathology, generally feel quieter; on the contrary, those who have no idea what IBD is, feel dismayed or apathy. Despite this, a commonly expressed feeling is represented by the relief of being able to name your suffering, although awareness of the possibility or reality of a life of management and treatment of IBD is shocking for many [32]. Increased knowledge has the potential to improve both physical and mental health outcomes during the transition from pediatric to adult care [38, 39]. The increasing knowledge of their body led the adolescents to try to identify triggers and patterns as well as to recognize the flare-ups. This situation gives them control over their illness, improving their decision-making [38], even if they can feel helpless or misunderstood. Finally, other factors that have been associated with greater knowledge of the disease include self-efficacy, health satisfaction, previous positive treatment experiences, and communication [38].

#### Physical needs

Loss of control impacts the feeling to be independent in adolescents. They suddenly experience feelings of embarrassment and alienation concerning both themselves and others, because they are dependent on constant access to toilet facilities. Moreover, they experience low energy and, if surgery is required, adolescents may suffer from pain and bloody diarrhea and are burned by anxiety. Some patients experience stomach pain, side effects such as weight gain or changes in facial form, and acne before and/or after the intervention [40].

#### Psychological needs

Young adults who are confronted with a chronic disease are often embarrassed and resentful because of the greater degree of physical and emotional dependence they experience at a time when, socially, they should be active, healthy, successful. These feelings of embarrassment can be very important to consider concerning even the earliest deeper love relationships that they find themselves experiencing [41]. Given that IBD is often diagnosed in young adulthood, a time when building intimate relationships and sexuality are particularly important, the difficulties relating to body image emerge with particular relevance at this stage. In particular, young women tend to overestimate the physical aspect in defining their personal and social values. As a result, being a woman with IBD is considered a risk factor for developing a negative body image and about 70% of female IBD patients report body dissatisfaction [42]. Moreover, depression and anxiety are the most common psychiatric problems in adolescents with IBD and can significantly impair their quality of life and school skills and even lead to suicide [43]. Moderate symptoms of anxiety and depression were found to be present at higher odds than severe symptoms, finding no significant differences between adolescents (10–17 years) and young adults (18–25 years). The active disease stage predicts the onset of depressive symptoms; at the same time, the female gender, together with disease activity and a shorter time since diagnosis predicts anxiety [44].

#### Need of protection, safeness

Unlike chronic diseases and/or disabilities diagnosed in early childhood, IBDs can be diagnosed on average around 15 years of age, subjecting adolescents to an accelerated transition path, and facilitating the experience of negative feelings such as, for example, embarrassment or vulnerability, and negative emotional experiences such as lack of control or social isolation [45, 46]. In particular, they are more prone to have low self-esteem, behavioral problems, alterations in the perception of body image, and difficulties in social skills. Further concerns concern not knowing what to expect in adult care, not appreciating their environment, not trusting new doctors and health professionals in general, and having to repeat their history, making sure that medical records are transferred to appropriately [47].

#### Relational and social needs

In an adult environment, you should have communication, negotiation, and assertiveness skills, so that you can assert your views effectively. Maturity and experience play an important role in developing these skills. Many adolescents suffering from IBD are intelligent, which is why their difficulty in self-promotion and social development may not be detected, with consequent delays in reaching developmental goals such as, for example, holidays without adults, work during school secondary and beyond, and falling in love for the first time [48]. Diagnosis and treatment of people with IBD pose challenges in participating in social activities, leading to the loss of opportunities to devote to leisure or school due to symptoms or frequent medical appointments [23, 27]. While most patients can achieve good symptom control, adherence to treatment prescriptions is quite poor. This is especially associated with some of the needs mentioned here as well as time constraints, medication side effects, poorly controlled disease activity and the perception that the drug is not working. Moreover, especially in adolescence, dysfunctional family and/or social relationships, social barriers and victimization may contribute to poor engagement and adherence in the treatment process [49]. Furthermore, it has been shown that patients aged 18 and over do not feel completely autonomous if the medicinal therapy to be taken is by injection or infusion or, again, in recognition of the need to resort to the support of a doctor or to be actively involved during a visit, especially if the symptoms are subtle. Patients are more likely to be successful when they have greater self-efficacy, which is the belief in their ability to complete specific tasks and achieve goals. To encourage mastery and self-efficacy, substantial areas of self-management can be broken down into smaller tasks [49, 50].

#### Need for gratitude, respect, and respect for your needs

Patient satisfaction with health care is recurrent and transversal in the different stages of people with IBD, and it is defined as the degree to which aspects of care considered important by the patient are met by experience. Different factors contribute to patient satisfaction and can be related to the patient characteristics, disease-related characteristics, and the health care provider. The main indicators of quality of care are synthesized in two categories: timing/logistics (i.e., usual time in a waiting room or consultation) and diagnosis/treatment (i.e. seeing the IBD doctor regularly, using maintenance therapy…) [51].

### Needs in transition

#### From a specialized pediatric team to a specialized adult team

Caring for a child with a chronic disease poses complex challenges in which the whole family is involved. The request for autonomy grows progressively during development and becomes pregnant in the transition to adulthood, during which the young person takes responsibility for his/her self: this step is also reflected in the transition between pediatric and adult care [52]. The transition is configured as a planned and gradual process through which the adolescent passes from a child-centered care system to an adult-centered one [53–55]. There are two main elements of this transition process that impact on its success: one is the passage of care from the primary caregiver (parent) to the patient, and the other is the transfer of responsibility from the pediatric gastroenterologist to one specialized in the care of adult subjects [56]. Improving transition preparation skills, including knowledge of the disease, has the potential to reduce gaps in continuity of care. Indeed, discontinuation of care put adolescents at risk for poorer health-related outcomes, including higher levels of non-adherence and non-engagement, potential loss of follow-ups [57], increased risk of relapse of the disease, and poorer overall health [58].

The transition process was conceptualized as having a beginning (the decision to switch to an “adult care”), an intermediate period (preparation for the transition, developing the necessary skills and behaviors that support self-care; effective health decision making and self-help), and end (effective transfer of care from a pediatric to adult care) [38].

The unpredictable course and the possible complications that characterize IBDs include a great variety of events whose nature can also be embarrassing with repercussions on the quality of life and some psychological, social, and physical aspects.

#### Psychology in transition

In the transition between adolescence and young adulthood, adolescents can express their disputed discomfort between the anxiety of leaving a well-known reference doctor and the need for independence and control over their illness. They need effective communication, education, and more information to facilitate their independent control.

Adolescents live relevant changes in their body, self-image, and identity. Therefore, they could feel as not treated as an adult, which can compromise the alliance on the ward. In this direction, they ask for advice on contraception, alcohol, smoking. Finally, a complex transition is often related to a dependent personality or anxiety, where parents use to manage the illness [59].

### The needs in adults with IBD

#### Needs of information

The information preferences are oriented to medical treatment, clinical manifestations, and surgery and the order of preference of methods for provision are as follows: face-to-face conversation, telephone, written dialogue (i.e., chats). The aspects on which an adult patient thinks to have fewer resources to access information are related to social rights (i.e., disability, financial aid management, tax benefits…), work right, risk of cancer, and/or of death. They prefer to have the opportunity to talk to the Gastroenterologist than to the General Practitioner or the Surgeon, consider a rate of satisfaction of 96% [60, 61]. In this life phase, a series of questions are also related to treatment failure and response with first-line and second-line biologic therapy with these two anti-TNFα agents [62]. IBD and, in particular, the CD can impact pregnancy and heredity. However, the decision to have children is negatively related to disease-related knowledge, and female patients are more worried about fertility issues than males [63]. Adults believe in the importance of receiving IBD education, trusting in its relevance improving quality of life and self-management [61] and coping strategies [64]. Finally, quality of life is considered more relevant than disease control [65].

#### Medical needs

Data about adherence and engagement about one's care process in adulthood are still controversial, which is why it is difficult to come to firm conclusions, especially when considering possible confounding factors such as random effects, disease activity, analyses and instruments adopted by studies, norms related to different countries, and patient selection. Some factors such as, example, age, gender or family history, duration of disease, or disease activity do not show homogeneous trends among studies. In addition, patient’s beliefs and/or specific situations such as pregnancy or future planning might impact treatment adherence differently [66, 67]. It is also relevant to note that, during periods of remission, fatigue could be one of the most important symptoms, and well-being and psychological functioning tend to be poorer, with significant impacts on quality of life and further clinical complications that intensify the cycle of inflammation and suffering, necessitating greater involvement in the engagement process [66].

Patients considering surgery for UC, express their need for informational shortcomings, especially related to the long-term effects of surgery, practicalities of daily living, and long-term support. Moreover, they frequently ask for peer support during and after the intervention [68].

#### Psychological needs

Jordan and colleagues found that extreme perceptions of the illness, being stressed, and adopting coping strategies focussed on the emotions, lead to worse mental health outcomes, a situation that is maintained when controlling the impact of clinical factors [69, 70]. They usually express stigma, feelings of shame, or of being dirty, due to the lack of control over their symptoms, powerlessness, humiliation, impacting on their self-image and self-esteem and resulting in isolation. Anxiety and depression tend to have higher rates in adults with IBD than in children and adolescents. This finding is associated with the longer duration of illness, the onset of complications often due to irreversible bowel damage as well as increased responsibilities and significant influences on daily life, especially work, that impact more than in childhood or adolescence. Indeed, unpredictable, and incurable course of the disease may impair the individual’s belief about self-control and self-efficacy. In addition, disease activity plays a significant role in the development of anxiety and/or depression [71]. Moreover, some studies describe the perceived lack of support during the recovery process, with a sense of being left after to adopt postoperatively. Rates of depression after surgery for UC range 11–17% and some participants report also difficulties related to body image and sexual life [68, 72, 73].

#### Social and work-related needs

According to Calvet et al. [74], in some cases, illness influences the kind of job performed or people lost or had rejected a job due to IBD. In particular, IBD patients with back/joint pain report significantly low productivity at work related to low quality of life [75, 76].

#### Support needs

People with IBDs report wanting to make sense of the illness, their emotions and coping strategies. To reach this aim, overwhelming participants look for psychological support from someone professional, with specialized knowledge of IBD, its symptoms, and emotional impact [70]. Nevertheless, the degree of psychological distress doesn’t correlate with seeking mental health support [77]. Klag and colleagues found that psychotherapy demand depends on fear of progression, quality of life, previous experience, smoking, and previous surgery [78].

Some patients define it as useful talking to other people with IBD in a group setting, because it helps to normalize the experience of the illness and reduce the sense of being alone or being misunderstood [70]. Social support is principally provided by family and close friends, health professionals, and self-help groups for people affected. The support received by the family helps to increase acceptance of the symptoms and to cope with them [79, 80]. On the other hand, the health care providers’ support is appreciated because they are supposed to know more about the symptoms, making patients feel confident and safe [81].

#### Practical needs

Most of the practical needs are related to the IBD symptoms. If considering the case of the CD, for example, the limitation of social life is represented by the organization of a trip, which is subject to the presence of toilets in the chosen destination area. The fear of incontinence and fear of unpredictability has important consequences on behavior: indeed, for many, this fear induces an avoidance of work, social and daily activities. Some persons try to cope with IBD, carrying potties and spare clothes, wearing adult diapers, or identifying toilets before traveling. They need to take into consideration extra time for traveling and control their bowel frequency [82]. At an institutional level, it becomes important to ensure continuity in the organization of public awareness campaigns (e.g., school, work), meetings that encourage the connection between IBDs services and those less specialized (e.g., General Practitioner), facilitation of bureaucratic procedures (e.g., exemptions, invalidity …). It could also be relevant to offer the possibility to a person suffering from IBD, thanks to a card or a special ticket, to access the toilets in public businesses.

Moreover, it is relevant to note that a self-prescribed dietary regimen is common among IBD patients as an attempt to improve their physical conditions and prolong remission intervals. Some foods, for instance, are recognized as a trigger for the induction of symptoms, leading to the risk of a serious nutritional deficiency [30, 83, 84]. Larussa and colleagues [84] noted that only 61% of the patients receive nutritional or dietary advice and 78% ask for more information. Therefore, dietary habits should be considered as relevant during the routine visits and some recommendations should be provided [85, 86].

#### Needs related to the future

Future planning is often hindered by increased anxiety and ambiguity about the disease trajectory. Chronic illness can impact the decision for bearing and raising children, because of worries about the implications of IBD [41]. According to Calvet et al. [74], some patients with UC report that illness influences their decision to have a baby (17.2%) or their ability to take care of children (40.7%), with higher percentages among women and in younger people [87].

#### Engagement needs

Disease management is a complex process where patients make decisions regarding care, symptoms, treatments, dietary modification, stress management, medications, and emotional components of their disease. The patients need information on both illness and the treatment options, including surgery, from an early stage. The decision-making process involves multiple factors, including disease status and the set of beliefs of the patients. Patients are often worried that, after surgery, the stoma bag would be seen or leak or smell. In addition, they are worried they would no longer be attractive to their partner. Sometimes, they are afraid about the impact of a stoma on their ability to bear a child or to have poor control over bowel function, limiting the possibility of a relationship or sporting [88]. All the elements arose above, including psychosocial factors such as social support, fear of surgery, fear of relapse, worries about self-image, provider recommendations contribute to the decision-making process [89, 90]. Finally, it is important to note that when the patient is actively involved in the decision-making process, the engagement is higher and the likelihood of a successful treatment outcome is maximized [91].

#### Recommendations and target interventions

Based on what was outlined in this scoping review, some recommendations and target interventions emerge for consideration at various stages of the life cycle as needs arise (Additional file 4: Table S4). The most recurrent recommendations in the different transitions and phases of the life cycle include those that aim to promote communication and the therapeutic alliance, intending to provide clear information that facilitates the decision-making process and the assumption of ownership of the care process. Also important are IBD education, psychological support offered to patients as well as parents and/or caregivers, and continuity of care.

## Discussions

This scoping review aimed to highlight the overriding psychological and social care and welfare needs of people with IBD as well as their evolutions over the life cycle. This has allowed us to distinguish and identify diverse needs in relation to different stages of the life cycle: children (psychological and needs to be accepted; physical and activity-related needs; nutrition-related needs; needs related to parental support; social needs); adolescents (needs to understand; physical needs; psychological needs; protection and safety needs; relational and social needs; gratitude, respect, and respect needs; in transition (related to transition from a pediatric to an adult team; psychological); adults (information needs; medical needs; psychological needs; social and work-related needs; practical needs; support needs; future-related needs; engagement needs), exploring factors that increase patient engagement. It allowed to identify and define the main research gaps in the literature about IBD’s needs and their evolution. In particular, this scoping review allowed us to note some elements of particular interests. First, it should be pointed out that chronic inflammatory bowel diseases, due to their complexity, require multi-perspective management, including psychological, practical, social, welfare, and existential goals. It is emphasized the need to take into consideration the psychological aspects in the management of IBD since the developmental age, identifying promptly patients and families who are at risk of distress. In this regard, it is desirable that the pediatric gastroenterologist would be accompanied by a psychologist since the first visit, also using specific validated psycho-diagnostic tools, as well as by nurses able to carry out actions of support. In this line, where necessary, provide nutritional advice that can give families adequate information on the diet. Although several transition clinic models currently exist, there is a paucity of data and a gap in the management of psychological, but also social, work-related, and practical needs caused by IBD and influencing the quality of life of patients and their caregivers [92, 93].

Secondly, based on the included articles, patients’ preferences and needs are relevant to sustain and facilitate engagement. Partnering with people with IBD throughout the lifespan with illness is a critical but essential step for shaping strategies and interventions. People with IBD are more willing to be actively involved in the health care process if can gain more information about the illness as well as the treatment options. The patient and caregivers’ engagement aim to foster greater accountability of the person with informed management of his/her illness, in a partnership with the health care providers. An individual effectively involved in his/her course of treatment, in addition to being more adherent to medical prescriptions is also more able to activate himself/herself properly at the first signs and symptoms of the disease and to benefit from the services offered by the more effectively and satisfactorily. This process can also enhance the IBD awareness and understanding in society, sustaining people with IBD in their daily struggle with practical, social, and psychological needs [94, 95].

Thirdly, the transition process to the management in adulthood of people with IBD might focus on the promotion of the patient's self-management and engagement, implementing the appropriate usage of monitoring tools [96, 97]. Due to the continued evolution of the needs, it could be important to build also a transitional IBD-Clinic managed by the collaboration between adult and pediatric professionals. This transitional IBD-Clinic might sustain the adolescent from the pediatric to the adult ward, encouraging a gradual assumption of responsibility for appropriate lifestyles, consolidating the levels of engagement already developed. It is essential to support the pediatric patient and his / her caregiver in the critical phases of the disease path: diagnosis (through psychological assessment and early psychological intervention), hospitalization, relapse of the disease, surgical intervention. It is also relevant to remember the multiple contexts in which the adolescent grows up: therefore, promoting a personalized network that connects the health dimension with daily life (i.e., school, family, peer group, sports clubs) according to the complexity and multi-component nature of the concept of health.

Furthermore, the presence of an extensive team (inside and outside the hospital) with varied and integrated skills, as well as the ability to stimulate the active role and participation in the care process need to be present along all the life cycle. Based on the previous literature, continuity of care is an important but less explored need. Maintaining the same doctor, and preferably the same care team is a guarantee of consistent work. In an already established therapeutic setting, digital technologies can be an effective support for maintaining therapeutic continuity and promoting the doctor-patient alliance [98]. In particular, the importance of sharing information between the specialist team and general practitioners is noted. In this direction, it also relevant to note the scarce attention paid to the transition to aging, where comorbidities and cognitive impairments might compromise more the clinical feature of the patient. It can therefore be noted that the needs emerged in this scoping review, and which characterize the different evolutionary phases, change in the life cycle of patients, requiring personalized and dedicated interventions depending on the life phase. Considering the recommendations and the target interventions, future research should explore the transition to school as well as those to remissions in children. On the other hand, in adolescence, the transition from secondary to high school, communication, medical knowledge and self-efficacy should be increased. Finally, in adulthood, future studies should pay attention to the promotion of common health problems not related directly to IBD such as pregnancy or fertility, always considering the relevance of self-efficacy, health literacy and communication.

Nonetheless, this study has several limitations. Firstly, we only included articles from after 2009. However, to limit this potential source of exclusion, additional material was identified following up the reference lists of articles included, without founding any previous consideration. Moreover, considering the last ten years gave us the possibility to pay attention to the contemporary needs, those more related to the last cultural and social changes. Nevertheless, every scoping review, does not formally evaluate the quality of evidence and often gathers information from a wide range of study designs and methods. Although a thorough search of the databases was performed, it is possible that this scoping review omitted relevant articles and did not cover the extent of the research available on this topic. Specifically, transitions in care, as well as need, are relatively broad concepts, lacking a universal definition, resulting in a lack of standardized terminology in the current literature databases. To minimize the possibility of missed articles, our search strategy was adapted for a variety of databases and included all key-words and Mesh headings relating to transitions in care and needs. A more rigorous definition of these terms is required. Furthermore, future studies are needed to help personalize and multi-professionals transition care practices, that pay attention to all the different needs related to IBD along the life circle, including all the transition phases and not only that to adulthood. Due to the few studies exploring patient perspectives during care transitions, especially during adulthood and towards aging, future research should seek to examine overall health and well-being during the different challenges of life. Future studies should also focus their attention on the differences that the remission and activity phases of the disease, considering the different evolutionary stages, allow to denote concerning the engagement process, aspect on which the available data are very superficial.

## Conclusions

This study provided an overview of the different kinds of needs and their evolutions in IBD along the life cycle. The findings can be adapted to inform the development of personalized health care processes as well as to identify, prioritize, and display gaps in the management of people with IBD. The literature shows the value of organizing the clinical intervention in a multidisciplinary perspective (through the enhancement of the IBD-Unit) and the broader active involvement of the patient and caregiver (s) (if requested by the patient and if necessary) through interventions to support and promote engagement. In this line, it is necessary to pay attention to the transition process to the management in adulthood of people with IBD, promoting the patient's self-management and engagement as well as his/her ability to manage medical, lifestyle self-management, and the health care process, also using appropriate monitoring tools such as self-assessment checklists. The information obtained from this review stressed the need to reflect on current policies regarding effective transitions in the health care process, utilization of services to improve IBD patient’s experiences. Finally, it highlighted the need for timely and accurate information-sharing and promotion of engagement along all the life cycle.

## Supplementary Information

**Additional file 1: Appendix S1.** Detailed search strategy for each search engine.

**Additional file 2: Table S2.** Main characteristics of the studies included.

**Additional file 3: Table S3.** Differences in care and needs divided considering the main theme and phase of the life cycle

**Additional file 4: Table S4.** Recommendations or targeted interventions to facilitate a transition

## Data Availability

The datasets used and/or analysed during the current study are available from the corresponding author on reasonable request.

## Abbreviations

IBD: Inflammatory Bowel Disease
UC: Ulcerative Colitis
CD: Crohn’s Disease
QoL: Quality of life
EEN: Exclusive Enteral Nutrition
PEN: Partial Enteral Nutrition
SCD: Specific carbohydrate diet
LFD: Lactose-free diet
CDED: Crohn’s disease exclusion diet

## References

[1] Lakatos PL (2006). Recent trends in the epidemiology of inflammatory bowel diseases: up or down?. World J Gastroenterol.

[2] Alatab S, Sepanlou SG, Ikuta K, Vahedi H, Bisignano C, Safiri S (2020). The global, regional, and national burden of inflammatory bowel disease in 195 countries and territories, 1990–2017: a systematic analysis for the Global Burden of Disease Study 2017. Lancet Gastroenterol Hepatol.

[3] Molodecky NA, Soon IS, Rabi DM, Ghali WA, Ferris M, Chernoff G (2012). Increasing incidence and prevalence of the inflammatory bowel diseases with time, based on systematic review. Gastroenterology.

[4] Ananthakrishnan AN (2015). Epidemiology and risk factors for IBD. Nat Rev Gastroenterol Hepatol.

[5] Cosnes J, Gowerrousseau C, Seksik P, Cortot A (2011). Epidemiology and natural history of inflammatory bowel diseases. Gastroenterology.

[6] Ng SC (2015). Emerging leadership lecture: inflammatory bowel disease in Asia: Emergence of a “Western” disease. J Gastroenterol Hepatol.

[7] Danese S, Fiocchi C (2011). Ulcerative colitis (supplementary appendix). N Engl J Med.

[8] Feuerstein JD, Cheifetz AS (2014). Ulcerative colitis: epidemiology, diagnosis, and management. Mayo Clin Proc.

[9] López-Sanromán A, Carpio D, Calvet X, Romero C, Cea-Calvo L, Juliá B (2017). Perceived emotional and psychological impact of ulcerative colitis on outpatients in Spain: UC-LIFE Survey. Dig Dis Sci.

[10] Rieder F, Bettenworth D, Ma C, Parker CE, Williamson LA, Nelson SA (2018). An expert consensus to standardise definitions, diagnosis and treatment targets for anti-fibrotic stricture therapies in Crohn’s disease. Aliment Pharmacol Ther.

[11] Van Tilburg MAL, Palsson OS, Whitehead WE (2013). Which psychological factors exacerbate irritable bowel syndrome? Development of a comprehensive model. J Psychosom Res.

[12] Lesage AC, Hagège H, Tucat G, Gendre JP (2011). Results of a national survey on quality of life in inflammatory bowel diseases. Clin Res Hepatol Gastroenterol.

[13] Sajadinejad MS, Asgari K, Molavi H, Kalantari M, Adibi P (2012). Psychological issues in inflammatory bowel disease: an overview. Gastroenterol Res Pract.

[14] Kelso M, Feagins LA (2018). Can smartphones help deliver smarter care for patients with inflammatory bowel disease?. Inflamm Bowel Dis.

[15] Kosinski LR, Brill J, Regueiro M (2017). Making a medical home for IBD patients. Curr Gastroenterol Rep.

[16] Weaver E, Szigethy E (2020). Managing pain and psychosocial care in IBD: a primer for the practicing gastroenterologist. Curr Gastroenterol Rep.

[17] Searle A, Bennett P (2001). Psychological factors and inflammatory bowel disease: a review of a decade of literature. Psychol Heal Med..

[18] Graff LA, Walker JR, Bernstein CN (2009). Depression and anxiety in inflammatory bowel disease: a review of comorbidity and management. Inflamm Bowel Dis.

[19] Kemp K, Griffiths J, Lovell K (2012). Understanding the health and social care needs of people living with IBD: a meta-synthesis of the evidence. World J Gastroenterol.

[20] McCombie AM, Mulder RT, Gearry RB (2013). How IBD patients cope with IBD: a systematic review. J Crohn’s Colitis.

[21] Tricco AC, Lillie E, Zarin W, O’Brien KK, Colquhoun H, Levac D (2018). PRISMA extension for scoping reviews (PRISMA-ScR): checklist and explanation. Ann Internal Med.

[22] Arksey H, O’Malley L (2005). Scoping studies: towards a methodological framework. Int J Soc Res Methodol Theory Pract..

[23] Zmeskalova D, Prasko J, Holubova M, Karaskova E, Marackova M, Slepecky M (2016). Unmet psychosocial needs in adolescents with inflammatory bowel disease. Neuroendocrinol Lett.

[24] Bokemeyer B, Hardt J, Hüppe D, Prenzler A, Conrad S, Düffelmeyer M (2013). Clinical status, psychosocial impairments, medical treatment and health care costs for patients with inflammatory bowel disease (IBD) in Germany: an online IBD registry. J Crohn’s Colitis.

[25] Dignass AU, Gasche C, Bettenworth D, Birgegård G, Danese S, Gisbert JP (2015). European consensus on the diagnosis and management of iron deficiency and anaemia in inflammatory bowel diseases. J Crohn’s Colitis.

[26] Mählmann L, Gerber M, Furlano RI, Legeret C, Kalak N, Holsboer-trachsler E (2017). Psychological wellbeing and physical activity in children and adolescents with inflammatory bowel disease compared to healthy controls. BMC Gastroenterol.

[27] Vedrana V, Bramhagen A-C, Idvall E, Wennick A (2017). Swedish children’s lived experience of ulcerative colitis. Gastroenterol Nurs.

[28] Pituch-Zdanowska A, Kowalska-Duplaga K, Jarocka-Cyrta E, Stawicka A, Dziekiewicz M, Banaszkiewicz A (2019). Dietary beliefs and behaviors among parents of children with inflammatory bowel disease. J Med Food.

[29] Schreiner P, Yilmaz B, Rossel J-B, Franc Y, Misselwitz B, Scharl M (2019). Vegetarian or gluten-free diets in patients with inflammatory bowel disease are associated with lower psychological well-being and a different gut microbiota, but no beneficial effects on the course of the disease. United Eur Gastroenterol J..

[30] Rocha R, Sousa UH, Reis TLM, Santana GO (2019). Nutritional status as a predictor of hospitalization in inflammatory bowel disease: a review. World J Gastrointest Pharmacol Ther..

[31] Miele E, Shamir R, Aloi M, Assa A, Braegger C, Bronsky J (2018). Nutrition in pediatric inflammatory bowel disease: a position paper on behalf of the porto inflammatory bowel disease group of the European society of pediatric gastroenterology, hepatology and nutrition. J Pediatr Gastroenterol Nutr.

[32] Kluthe C, Isaac DM, Hiller K, Carroll M, Hons B, Fracp M (2018). Qualitative analysis of pediatric patient and caregiver perspectives after recent diagnosis with inflammatory bowel disease. J Pediatr Nurs.

[33] Lindström C, Åman J, Anderzén-Carlsson A, Lindahl NA (2016). Group intervention for burnout in parents of chronically ill children—a small-scale study. Scand J Caring Sci.

[34] Otto C, Tárnok A, Erős A, Szakács Z, Vincze Á, Farkas N (2019). Planned transition of adolescent patients with inflammatory bowel disease results in higher remission rates. J Pediatr Nurs.

[35] Vejzovic V, Bramhagen AC, Idvall E, Wennick A (2018). Swedish children’s lived experience of ulcerative colitis. Gastroenterol Nurs.

[36] Cervesi C, Battistutta S, Martelossi S, Ronfani L, Ventura A (2013). Health priorities in adolescents with inflammatory bowel disease: physicians’ versus patients’ perspectives. J Pediatr Gastroenterol Nutr.

[37] Benchimol EI, Walters TD, Kaufman M, Frost K, Fiedler K, Chinea Z (2011). Assessment of knowledge in adolescents with inflammatory bowel disease using a novel transition tool. Inflamm Bowel Dis.

[38] Gumidyala AP, Plevinsky JM, Poulopoulos N, Kahn SA, Walkiewicz D, Greenley RN (2017). What teens do not know can hurt them: an assessment of disease knowledge in adolescents and young adults with IBD. Inflamm Bowel Dis.

[39] Moradkhani A, Kerwin L, Dudley-Brown S, Tabibian JH (2011). Disease-specific knowledge, coping, and adherence in patients with inflammatory bowel disease. Dig Dis Sci.

[40] Olsen IØ, Jensen S, Larsen L, Sørensen EE (2016). Adolescents’ lived experiences while hospitalized after surgery for ulcerative colitis. Gastroenterol Nurs.

[41] Nutting R, Grafsky EL (2018). Crohn’s disease and the young couple: an interpretative phenomenological analysis. Contemp Fam Ther.

[42] Trindade IA, Ferreira C, Pinto-Gouveia J (2017). The effects of body image impairment on the quality of life of non-operated Portuguese female IBD patients. Qual Life Res.

[43] Zhang CK, Hewett J, Hemming J, Grant T, Zhao H, Abraham C (2013). The influence of depression on quality of life in patients with inflammatory bowel disease. Inflamm Bowel Dis.

[44] van den Brink G, Stapersma L, Vlug LE, Rizopolous D, Bodelier AG, van Wering H (2018). Clinical disease activity is associated with anxiety and depressive symptoms in adolescents and young adults with inflammatory bowel disease. Aliment Pharmacol Ther.

[45] Gray WN, Resmini AR, Baker KD, Holbrook E, Morgan PJ, Ryan J (2015). Concerns, barriers, and recommendations to improve transition from pediatric to adult IBD care: perspectives of patients, parents, and health professionals. Inflamm Bowel Dis.

[46] Gray WN, Reed-Knight B, Morgan PJ, Holbrook E, Kugathasan S, Saeed SA (2018). Multi-site comparison of patient, parent, and pediatric provider perspectives on transition to adult care in IBD. J Pediatr Nurs.

[47] Reiss JG, Gibson RW, Walker LR (2017). Health care transition: youth, family, and provider perspectives. Pediatrics.

[48] Hummel TZ, Tak E, Maurice-Stam H, Benninga MA, Kindermann A, Grootenhuis MA (2013). Psychosocial developmental trajectory of adolescents with inflammatory bowel disease. J Pediatr Gastroenterol Nutr.

[49] Adler J, Sc M (2016). Transition readiness in pediatric patients with inflammatory bowel disease: a patient survey of self-management skills. J Pediatr Gastroenterol Nutr.

[50] De Silva PSA, Fishman LN (2014). Transition of the patient with IBD from pediatric to adult care-an assessment of current evidence. Inflamm Bowel Dis.

[51] Otto-Sobotka F, Peplies J, Timmer A (2019). Modeling determinants of satisfaction with health care in youth with inflammatory bowel disease part 2: semiparametric distributional regression. Clin Epidemiol.

[52] Schwartz LA, Tuchman LK, Hobbie WL, Ginsberg JP (2011). A social-ecological model of readiness for transition to adult-oriented care for adolescents and young adults with chronic health conditions. Child Care Health Dev.

[53] Blum RW, Garell D, Hodgman CH, Jorissen TW, Okinow NA, Orr DP (1993). Transition from child-centered to adult health-care systems for adolescents with chronic conditions. A position paper of the Society for Adolescent Medicine. J Adolesc Health.

[54] Cooley WC (2013). Adolescent health care transition in transition. JAMA Pediatr.

[55] Campbell F, Biggs K, Aldiss SK, O’Neill PM, Clowes M, Mcdonagh J (2016). Transition of care for adolescents from paediatric services to adult health services. Cochrane Database Syst Rev.

[56] Afzali A, Wahbeh G (2017). Transition of pediatric to adult care in inflammatory bowel disease: is it as easy as 1, 2, 3?. World J Gastroenterol.

[57] Whitfield EP, Fredericks EM, Eder SJ, Shpeen BH, Adler J (2015). Transition readiness in pediatric patients with inflammatory bowel disease. J Pediatr Gastroenterol Nutr.

[58] Bollegala N, Brill H, Marshall JK (2013). Resource utilization during pediatric to adult transfer of care in IBD. J Crohn’s Colitis.

[59] McCartney S (2011). Inflammatory bowel disease in transition: challenges and solutions in adolescent care. Front Gastroenterol.

[60] Catalán-Serra I, Huguet-Malavés JM, Mínguez M, Torrella E, Paredes JM, Vázquez N (2015). Information resources used by patients with inflammatory bowel disease: satisfaction, expectations and information gaps. Gastroenterol Hepatol.

[61] McDermott E, Healy G, Mullen G, Keegan D, Byrne K, Guerandel A (2018). Patient education in inflammatory bowel disease: a patient-centred, mixed methodology study. J Crohn’s Colitis.

[62] Gordon JP, Mcewan PC, Maguire A, Sugrue DM, Puelles J (2015). Characterizing unmet medical need and the potential role of new biologic treatment options in patients with ulcerative colitis and Crohn’s disease: a systematic review and clinician surveys. Eur J Gastroenterol Hepatol.

[63] Wu Q, Zhong J (2018). Disease-related information requirements in patients with Crohn’s disease. Patient Prefer Adherence.

[64] Wardle RA, Mayberry JF (2014). Patient knowledge in inflammatory bowel disease: the Crohn’s and colitis knowledge score. Eur J Gastroenterol Hepatol.

[65] van Deen WK, Nguyen D, Duran NE, Kane E, van Oijen MGH, Hommes DW (2017). Value redefined for inflammatory bowel disease patients: a choice-based conjoint analysis of patients’ preferences. Qual Life Res.

[66] Trindade IA, Ferreira C, Pinto-Gouveia J (2018). The longitudinal effects of emotion regulation on physical and psychological health: a latent growth analysis exploring the role of cognitive fusion in inflammatory bowel disease. Br J Health Psychol.

[67] Albenberg L, Brensinger CM, Wu Q, Gilroy E, Kappelman MD, Sandler RS (2019). A diet low in red and processed meat does not reduce rate of Crohn’s disease flares. Gastroenterology.

[68] Baker DM, Lee MJ, Jones GL, Brown SR, Lobo AJ (2018). The Informational needs and preferences of patients considering surgery for ulcerative colitis: results of a qualitative study. Inflamm Bowel Dis.

[69] Jordan C, Sin J, Fear NT, Chalder T (2016). A systematic review of the psychological correlates of adjustment outcomes in adults with inflammatory bowel disease. Clin Psychol Rev.

[70] Jordan C, Ohlsen R, Hayee B, Chalder T (2018). A qualitative study exploring the experience of people with IBD and elevated symptoms of anxiety and low mood and the type of psychological help they would like. Psychol Health.

[71] Byrne G, Rosenfeld G, Leung Y, Qian H, Raudzus J, Nunez C (2017). Prevalence of anxiety and depression in patients with inflammatory bowel disease. Can J Gastroenterol Hepatol.

[72] Brown C, Gibson PR, Hart A, Kaplan GG, Kachroo S, Ding Q (2015). Long-term outcomes of colectomy surgery among patients with ulcerative colitis. Springerplus.

[73] Ananthakrishnan AN, Gainer VS, Cai T, Perez RG, Cheng SC, Savova G (2013). Similar risk of depression and anxiety following surgery or hospitalization for crohn’s disease and ulcerative colitis. Am J Gastroenterol.

[74] Calvet X, Argüelles-Arias F, López-Sanromán A, Cea-Calvo L, Juliá B, De Santos CR (2018). Patients’ perceptions of the impact of ulcerative colitis on social and professional life: results from the UC-LIFE survey of outpatient clinics in Spain. Patient Prefer Adherence.

[75] van der Have M, Brakenhoff LKPM, van Erp SJH, Kaptein AA, Leenders M, Scharloo M (2015). Back/joint pain, illness perceptions and coping are important predictors of quality of life and work productivity in patients with inflammatory bowel disease: a 12-month longitudinal study. J Crohns Colitis.

[76] Haapamäki J, Heikkinen E, Sipponen T, Roine RP, Haapam J (2018). The impact of an adaptation course on health-related quality of life and functional capacity of patients with inflammatory bowel disease functional capacity of patients with inflammatory bowel disease. Scand J Gastroenterol.

[77] Knowles S, Andrews JM, Porter A, Hons B (2018). Predictors of impaired mental health and support seeking in adults with inflammatory bowel disease: an online survey. Gastroenterol Nurs.

[78] Klag T, Mazurak N, Fantasia L, Schwille-Kiuntke J, Kirschniak A, Falch C (2017). High demand for psychotherapy in patients with inflammatory bowel disease. Inflamm Bowel Dis.

[79] Lahat A, Neuman S, Eliakim R, Ben-Horin S (2014). Partners of patients with inflammatory bowel disease: how important is their support?. Clin Exp Gastroenterol.

[80] Chiapponi C, Witt M, Dlugosch GE, Gu V, Gulberg V, Siebeck M (2016). The perception of physician empathy by patients with inflammatory bowel disease. PLoS ONE.

[81] Garcia-Sanjuan S, Lillo-Crespo M, Sanjuan-Quiles A, Gil-Gonzalez D, Richart-Martinez M (2016). Life experiences of people affected by Crohn’s disease and their support networks: scoping review. Clin Nurs Res.

[82] Kemp K, Griffiths J, Campbell S, Lovell K (2013). An exploration of the follow-up up needs of patients with inflammatory bowel disease. J Crohns Colitis.

[83] De Vries JHM, Dijkhuizen M, Tap P, Witteman BJM (2019). Patient’s dietary beliefs and behaviours in inflammatory bowel disease. Dig Dis.

[84] Larussa T, Suraci E, Marasco R, Imeneo M, Abenavoli L, Luzza F (2019). Self-prescribed dietary restrictions are common in inflammatory bowel disease patients and are associated with low bone mineralization. Medicina (Kaunas).

[85] Tomar SK, Kedia S, Upadhyay AD, Bopanna S, Yadav DP, Goyal S (2017). Impact of dietary beliefs and practices on patients with inflammatory bowel disease: an observational study from India. J Gastroenterol Hepatol.

[86] Hou JK, Lee D, Lewis J (2014). Diet and inflammatory bowel disease: review of patient-targeted recommendations. Clin Gastroenterol Hepatol.

[87] Ghorayeb J, Branney P, Selinger CP, Madill A (2018). When your pregnancy echoes your illness: transition to motherhood with inflammatory bowel disease. Qual Health Res.

[88] Dibley L, Czuber-dochan W, Wade T, Duncan J, Burch J, Warusavitarne J (2018). Patient decision-making about emergency and planned stoma surgery for IBD: a qualitative exploration of patient and clinician perspectives. Inflamm Bowel Dis.

[89] Kamp KJ, Brittain K (2018). Factors that influence treatment and non-treatment decision making among individuals with inflammatory bowel disease: an integrative review. Patient.

[90] Siegel CA, Lofland JH, Naim A, Gollins J, Walls DM, Rudder LE (2015). Novel statistical approach to determine inflammatory bowel disease: patients’ perspectives on shared decision making. Patient Patient-Centered Outcomes Res..

[91] Mahlich J, Sruamsiri R, Matsuoka K, Sruamsiri R (2017). Shared decision making and treatment satisfaction in Japanese patients with inflammatory bowel disease. Dig Dis.

[92] Sweeney L, Norton C (2018). Systematic review: psychosocial factors associated with pain in inflammatory bowel disease. Aliment Pharmacol Ther.

[93] Clarke T, Lusher J (2016). Transitioning patients with inflammatory bowel disease (IBD) from adolescent to adult services: a systematic review. Frontline Gastroenterol..

[94] Liang L, Cako A, Urquhart R, Straus SE, Wodchis WP, Baker GR (2018). Patient engagement in hospital health service planning and improvement: a scoping review. BMJ Open.

[95] Barello S, Graffigna G (2015). Patient engagement in healthcare: pathways for effective medical decision making. Neuropsychol Trends.

[96] Rohatinsky N, Risling T, Kumaran M, Hellsten LAM, Thorp-Froslie N (2018). Healthcare transition in pediatrics and young adults with inflammatory bowel disease: a scoping review. Gastroenterol Nurs.

[97] van Rheenen PF, Aloi M, Biron IA, Carlsen K, Cooney R, Cucchiara S (2017). European Crohn’s and colitis organisation topical review on transitional care in inflammatory bowel disease. J Crohn’s Colitis.

[98] Peris MA, Del Hoyo J, Bebia P, Faubel R, Barrios A, Bastida G (2015). Telemedicine in inflammatory bowel disease: opportunities and approaches. Inflamm Bowel Dis.

